# Subacute Octahydro-1,3,5,7-tetranitro-1,3,5,7-tetrazocine Exposure Induces Neurobehavioral Deficits and Hippocampal Demyelination in Mice

**DOI:** 10.3390/toxics14070605

**Published:** 2026-07-11

**Authors:** Xiaoqiang Lv, Cunzhi Li, Yinan Zhang, Qian Luo, Ting Gao, Hui Deng, Huan Li, Xinying Peng, Jiachen Shen, Siqi Liu, Junhong Gao, Zhiyong Liu

**Affiliations:** 1 Toxicology Research Center, Institute for Hygiene of Ordnance Industry, Xi’an 710065, China; 2 Xi’an Key Laboratory of Toxicology and Biological Effect, Xi’an 710065, China; 3 Laboratory for Bone Metabolism, Xi’an Key Laboratory of Special Medicine and Health Engineering, Key Laboratory for Space Biosciences and Biotechnology, Research Center for Special Medicine and Health Systems Engineering, School of Life Sciences, Northwestern Polytechnical University, Xi’an 710072, China; 4 College of Public Health, Ningxia Medical University, Yinchuan 750004, China

**Keywords:** HMX, neurotoxicity, hippocampal demyelination, learning and memory deficits, proteomics

## Abstract

Octahydro-1,3,5,7-tetranitro-1,3,5,7-tetrazocine (HMX) is a nitramine explosive widely used in military and industrial fields. While emerging evidence suggests the neurotoxicity of HMX, the mechanisms underlying central nervous system (CNS) damage remain largely unknown. In the present study, we established a mouse model of 28-day subacute HMX exposure to explore HMX-induced neurotoxicity and underlying mechanisms in vivo. Behavioral assessments revealed that HMX increased spontaneous locomotor activity and central exploration in the open field test, and reduced immobility time in the forced swimming test, indicating abnormal emotional regulation. The Morris water maze further demonstrated impaired hippocampus-dependent spatial learning and memory in HMX-treated mice, as evidenced by prolonged platform latency. Histopathological analysis showed hippocampal demyelination in HMX-treated mice, accompanied by downregulation of myelin structural proteins (MBP, PLP1) and oligodendrocyte lineage proteins (OLIG2, CNPase). Additionally, proteomic analysis identified 173 differentially expressed proteins in the HMX-exposed hippocampus, which were enriched in myelination, synaptic transmission and neuroactive ligand–receptor interaction pathways. Collectively, our findings demonstrate that subacute HMX exposure induces behavioral deficits and demyelination in mice hippocampus, providing a novel mechanistic insight into HMX neurotoxicity and a theoretical basis for occupational health protection against HMX exposure.

## 1. Introduction

Energetic materials are high-energy-density chemicals containing reactive functional groups such as nitro, azide, and hydrazine, which are indispensable for military, aerospace, and mining industries [1]. With widespread production and application, the potential biotoxicity and environmental risks of energetic materials have attracted increasing global attention [2,3,4]. Among nitramine explosives, cyclotrimethylenetrinitramine (RDX) and hexanitrohexaazaisowurtzitane (CL-20) have well-characterized severe neurotoxicity targeting the central nervous system. For instance, CL-20 initiates neurotoxicity by noncompetitively blocking the ligand-gated GABAA receptor ion channel, while RDX triggers epileptic seizures in humans [5,6].

As another typical nitramine explosive, octahydro-1,3,5,7-tetranitro-1,3,5,7-tetrazocine (HMX) is extensively utilized in mining and military industries worldwide [7]. Although acute toxicity studies have shown that HMX has a relatively high median lethal dose (LD_50_: 2000 mg/kg in mice, 6500 mg/kg in rats) compared to other nitramines, accumulating evidence indicates that HMX exposure can cause persistent CNS damage [8]. Preclinical studies have demonstrated that HMX accumulates in the brain and induces seizures, convulsions, and hyperactivity in various animal species, including rabbits, reptiles, and rodents [9,10,11,12]. In industrial production, explosive charging and waste disposal processes, HMX mainly enters the human body via inhalation of dust and dermal contact, and accidental ingestion through hand-to-mouth contact also occurs. Workplace air monitoring for HMX can be conducted using established national standard methods (GBZ/T 160.80-2004 in China) [13]. Moreover, regarding occupational exposure limits for HMX, China has issued the relevant standard (GB/Z 2.1-2019 in China) [14], in which the permissible concentration–time weighted average (PC-TWA) is set at 2 mg/m^3^ and the permissible concentration–short-term exposure limit (PC-STEL) at 4 mg/m^3^. Epidemiological investigations further revealed that occupational HMX exposure is associated with a range of neurological symptoms in workers, such as emotional disturbances, attention deficits, memory impairment, and altered peripheral nerve conduction velocity [15]. Although the neurotoxicity of HMX has been repeatedly observed, the underlying mechanism remains poorly understood.

Our previous in vitro study demonstrated that HMX exerts significant neurotoxicity in differentiated PC12 cells, characterized by calcium overload, increased ROS production, and dysregulated expression of neurotrophic factors and glutamate receptors [16]. Building on these findings, the present study aimed to investigate the in vivo neurotoxic effects of subacute HMX exposure and elucidate the underlying mechanisms using a mouse model. We performed comprehensive behavioral assessments to evaluate emotional and cognitive functions, combined with histopathological, immunohistochemical, and proteomic analyses to explore the molecular basis of HMX-induced neurotoxicity. Our results reveal that HMX induces hippocampal demyelination and inhibits oligodendrocyte maturation, which contributes to the observed neurobehavioral deficits. These findings advance our understanding of HMX neurotoxicity and have important implications for the prevention and treatment of HMX-induced neurological disorders.

## 2. Materials and Methods

### 2.1. Animals and Treatment

Eight-week-old male C57BL/6 mice were obtained from Beijing Sibeifu Biotechnology Co., Ltd. (Beijing, Chian). (SCXK (Jing) 2021-0006). Only male mice were used in this study to eliminate potential confounding effects of the estrous cycle on behavioral and neurochemical outcomes. Animals were housed in a specific pathogen-free (SPF) animal room under a 12 h light/dark cycle with free access to food and water. Housing conditions were maintained at 23 ± 2 °C and 50 ± 10% relative humidity. All animal experiments were approved by the Animal Care and Use Committee of Institute for Hygiene of Ordnance Industry (approval number: ICAUC202510) and conducted in strict accordance with the 3R principle to minimize animal discomfort.

HMX (purity ≥ 98%) was provided by Xi’an Modern Chemistry Research Institute (Xi’an, Chian). Sodium carboxymethyl cellulose (CMC-Na, analytical grade) was purchased from Sinopharm Chemical Reagent Co., Ltd. (Shanghai, Chian). Sodium carboxymethyl cellulose (CMC-Na, 1% *w*/*v*) was used as the suspending agent for HMX due to its excellent suspending properties and well-established safety profile in rodent toxicology studies. The samples solution was then ultrasonicated for 30 min to form a homogeneous suspension for intragastric (i.g.) administration. The control group received an equivalent volume of 1% CMC-Na solution to control for both vehicle effects and gavage procedure-related stress. Mice were randomly divided into four groups (*n* = 10 per group): control group (1%CMC-Na), or low-dose HMX group (40 mg/kg), medium-dose HMX group (100 mg/kg), and high-dose HMX group (250 mg/kg). The dose selection was based on previous acute toxicity studies and preliminary experiments showing no mortality or severe acute toxicity at these doses. The three dose levels of HMX, 1/50, 1/20, and 1/8 of the mouse oral LD_50_, respectively, include the LOAEL (100 mg/kg) and cover a gradient from NOAEL to clear neurotoxic effects, consistent with the standard design of subacute toxicity studies. Mice were administered once daily by gavage at a volume of 10 mL/kg body weight for 28 consecutive days. Although inhalation and dermal contact are the primary routes of occupational HMX exposure, the intragastric route was selected to ensure precise dose control and reliable dose–response relationship. Moreover, oral gavage is consistent with standard subacute toxicity test protocols and allows direct comparability with the extensive existing HMX toxicology literature. Body weight was measured weekly to adjust the administration volume.

### 2.2. Open Field Test

Behavioral tests were performed 24 h after the last HMX administration in the following order: open field test, forced swimming test, and Morris water maze test. This order was chosen to minimize the carryover effects of more stressful tests on subsequent assessments. All behavioral tests were conducted between 08:00 and 16:00 in a quiet, dimly lit room. Mice were acclimated to the testing room for at least 2 h before each test.

The open field test (OFT) was performed to evaluate spontaneous locomotor activity and anxiety-like behaviors in mice. The experimental apparatus consisted of a 50 cm × 50 cm × 35 cm (L × W × H) square box. At the start of the trial, each mouse was placed individually in the central area of the box, and its behavior were recorded for 5 min using a video tracking system (TSE System, Thuringia, Germany). Behavioral parameters including total traveled distance, central area distance and central zone entries were analyzed blindly. After each trial, the apparatus was thoroughly cleaned with 75% ethanol to remove residual odor and excreta, which avoided interference with subsequent tests.

### 2.3. Forced Swimming Test

The forced swimming test (FST) was applied to evaluate the degree of desperation and helplessness in mice. Rodents instinctively struggle to escape from the water environment, and the immobility reflects behavioral despair. The experimental apparatus was an open container filled with water maintained at 23 ± 1 °C. On the day of the test, mice were individually placed into the container for a 6 min free swimming trial. During data analysis, the immobility time of mice during the last 5 min of the behavior was blindly recorded as a measure of despair-like behavior.

### 2.4. Morris Water Maze

The Morris water maze (MWM) is a classic behavioral paradigm for assessing hippocampus-dependent spatial learning and memory in rodents. Rodents use visual cues placed around the pool to form a spatial reference memory of the platform location, reflecting their abilities in learning acquisition and retrieval. The experimental apparatus was of a circular pool 100 cm in diameter and 60 cm deep, filled with water at 23 ± 1 °C. A blue curtain surrounded the pool, and distinct shapes attached to the curtain served as spatial cues. Before testing, mice were acclimated to the testing room for at least 2 h. The entire experiment consisted of a place navigation phase (days 1–5) and a spatial probe phase (day 6). During place navigation, an escape platform was hidden below the water surface. Mice were released from each of the four quadrants and allowed to search for the platform for up to 60 s. When the mouse failed to locate the platform within 60 s, it was guided to the platform, and the latency was recorded as 60 s. During the spatial probe, the platform was removed. Each mouse was released from the quadrant opposite the original platform position and allowed to swim freely. The latency to cross the former platform location and the swimming trajectory were recorded.

### 2.5. Hematoxylin–Eosin Staining

At 24 h after the last behavioral test, mice were deeply anesthetized with isoflurane and euthanized by cervical dislocation. Mouse brain tissues were then collected and fixed overnight in 4% paraformaldehyde (Biosharp, Anhui, China, BL539A). After dehydration through graded ethanol solutions, the tissues were embedded in paraffin and sectioned at 6 μm thickness. The slides were air-dried and then baked at 70 °C for 60 min. Subsequently, the sections were deparaffinized in xylene and rehydrated through the graded ethanol solution. Nuclei were stained with hematoxylin (Baso, Zhuhai, China, BA4041) for 5 min, followed by differentiation in 1% acid alcohol for 3 s and bluing in 1% ammonia water for 5 s. Cytoplasm was stained with eosin (Baso, Zhuhai, China, BA4024) for 5 min. Finally, the sections were dehydrated through graded ethanol, cleared in xylene, and mounted with neutral balsam.

### 2.6. Nissl Staining

Paraffin sections (6 μm) were deparaffinized in xylene and rehydrated through graded ethanol solutions, then cleaned with PBS. The sections were incubated in 1% cresyl violet solution at 56 °C in a water bath for 1 h. After incubation, the slides were rinsed with water and differentiated in an ethanol solution for 1 min. Finally, the sections were cleared in xylene and mounted with neutral balsam.

### 2.7. Luxol Fast Blue Staining

Luxol Fast Blue (LFB), a copper-phthalocyanine dye, specifically stains the myelin sheath in neural tissue. Stained myelin appears bright blue, while the background is colorless to light blue. After rehydration, paraffin sections were incubated in LFB solution (Sigma-Aldrich, St. Louis, MO, USA, S3382) at 56 °C in a water bath for 2 h. Excess staining solution was removed by washing with 95% ethanol, followed by a 1 min rinse with water. The sections were then treated with 1% lithium carbonate solution (Sigma-Aldrich, St. Louis, MO, USA, 255823) and 75% ethanol for 15 s each, followed by another 1 min water rinse. Subsequent dehydration, clearing, and mounting steps were the same as those used for H&E staining. After the stain was completed, sections were photographed under a microscope.

### 2.8. Immunofluorescence Staining

Mouse brain sections were deparaffinized in xylene, rehydrated through a graded ethanol solution, and washed with PBS (Biosharp, Anhui, China, BL601A). Antigen retrieval was performed using sodium citrate buffer (Solarbio, Beijing, China, C1032) with microwave heating at medium power for 10 min. The sections were then blocked and permeabilized for 40 min at room temperature in PBS containing 5% goat normal serum (Bioss, Beijing, China, C01-03001) and 0.3% Triton X-100 (Biosharp, Anhui, China, BL935B). Subsequently, the slices were incubated with primary antibody against MBP (Proteintech, Chicago, IL, USA, 1:250, 10458-1-AP), Myelin PLP1 (Abcam, Cambridge, UK, 1:250, ab254363), CNPase (Proteintech, Chicago, IL, USA, 1:200, 13427-1-AP) and OLIG2 (Proteintech, Chicago, IL, USA, 1:500, 13999-1-AP) diluted in antibody diluent solution (NCM Biotech, WB500D) overnight at 4 °C. After washing with PBS for 30 min, the sections were incubated with alexa Fluor™594 (Thermo Fisher Scientific, Waltham, MA, USA, 1:500, A21207) and 488 (Thermo Fisher Scientific, Waltham, MA, USA, 1:400, A21206) conjugated anti-rabbit secondary antibody for 1 h at room temperature in the dark. Finally, nuclei were counterstained with DAPI solution (Proteintech, Chicago, IL, USA, PR30021) under dark conditions. Images were acquired using a Leica SP8 laser confocal microscope (Leica, Wetzlar, Germany).

For quantification of immunofluorescence staining, hippocampal subregions (CA1, CA3, and DG) were identified based on standard anatomical landmarks. For each animal, three non-consecutive sections were randomly selected, and the mean fluorescence intensity of each hippocampal subregion was quantified across these sections. To ensure the accuracy of individual animal data, and given that the hippocampus is a bilateral structure, subregional data from the left and right hippocampus were analyzed separately. Images were acquired under identical exposure settings for all samples within each antibody staining batch. The ‘protein-positive area’ was defined as the percentage of the total ROI area occupied by specific immunofluorescence signal above a standardized threshold, calculated using ImageJ (version 2.0.0). All quantifications were performed by investigators blinded to group allocation.

### 2.9. Proteomics Sequencing

Hippocampal tissues were dissected from control and high-dose HMX mice (*n* = 5 per group) and stored at −80 °C until use. Tissues were homogenized in SDT lysis buffer containing protease inhibitors. The homogenates were sonicated on ice and then centrifuged at 14,000× *g* for 15 min at 4 °C. The supernatant was collected, and protein concentration was determined using the BCA protein assay kit (Beyotime, Shanghai, China).

For each sample, 100 μg of protein was reduced with 10 mM DTT at 56 °C for 30 min, alkylated with 50 mM iodoacetamide in the dark for 30 min, and then precipitated with four volumes of cold acetone at −20 °C overnight. The protein pellet was washed twice with acetone, air-dried, and resuspended in 50 mM triethylammonium bicarbonate (TEAB) buffer. Proteins were digested with trypsin (Promega Corporation. Madison, WI, USA) at an enzyme-to-substrate ratio of 1:50 (*w*/*w*) at 37 °C for 16 h. The resulting peptides were desalted using C18 SPE columns (Thermo Fisher Scientific, Waltham, MA), eluted with 70% acetonitrile containing 0.1% formic acid, and lyophilized.

Peptides were reconstituted in 0.1% formic acid and analyzed using a Vanquish Neo nano-UHPLC system coupled to a Thermo Orbitrap Astral mass spectrometer (Thermo Fisher Scientific, Waltham, MA). Peptides were separated on a PepMap Neo C18 column at a flow rate of 300 nL/min using a linear gradient of 2–30% acetonitrile in 0.1% formic acid over 90 min. Mass spectrometry data were acquired in data-independent acquisition (DIA) mode with the following parameters: electrospray voltage 2.0 kV, ion transfer tube temperature 290 °C, MS1 scan range *m*/*z* 380–980 at 240,000 resolution, 300 consecutive isolation windows of 2 Th, normalized collision energy 25%, and MS2 scan range *m*/*z* 150–2000 at 80,000 resolutions.

Raw data were processed using DIA-NN software (version 1.8) against the UniProt mouse proteome database. The search parameters included: precursor mass tolerance 10 ppm, fragment mass tolerance 0.02 Da, fixed modification of cysteine carbamidomethylation, variable modifications of protein N-terminal acetylation and methionine oxidation, maximum two missed cleavages, and false discovery rate (FDR) < 1% at both peptide and protein levels. Differentially expressed proteins (DEPs) were defined as those with a fold change > 1.5 and a *p*-value < 0.05 (Student’s *t*-test). Functional enrichment analysis of DEPs was performed using Gene Ontology (GO) and Kyoto Encyclopedia of Genes and Genomes (KEGG) databases.

### 2.10. Statistical Analysis

GraphPad Prism 8 (GraphPad, La Jolla, CA, USA) was used for statistical analysis. Significant differences were evaluated by one-way ANOVA. All data were presented as mean ± standard error of the mean (SEM), and values of *p* less than 0.05 were considered significant.

## 3. Results

### 3.1. Decreased Anxiety-like and Despair-like Behaviors in HMX-Treated Mice

**Figure 1:**
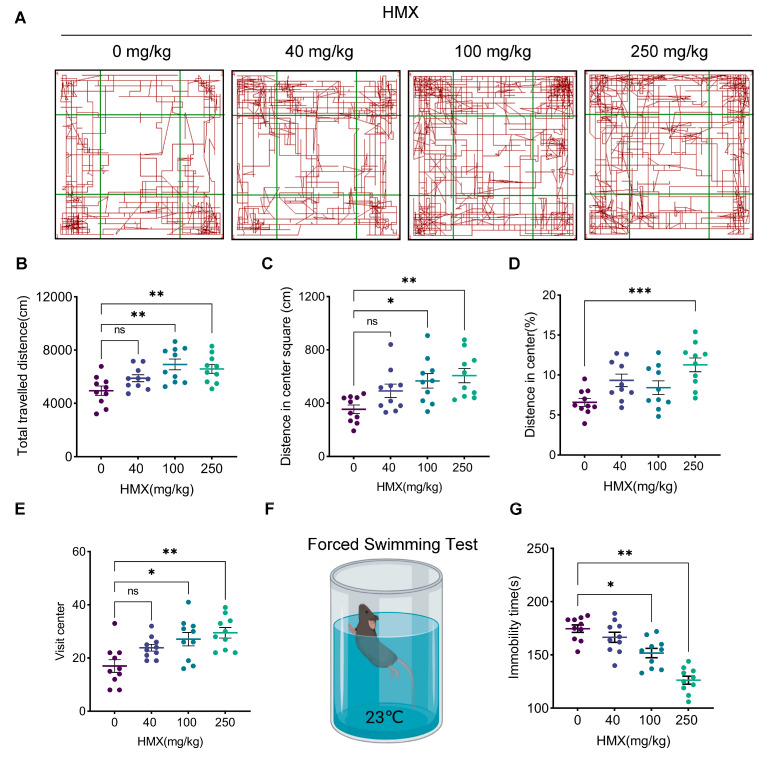
Abnormal anxiety-like and despair-like behaviors in HMX-treated mice. (**A**) Representative track path of all mice in the open field test, red lines indicate the locomotor trajectories of animals, and green lines denote the boundaries of the defined regions. (**B**) Quantification of the total distance traveled in the open field test. (**C**,**D**) Significant reduction in distance in center square and percentage of distance in HMX-treated mice. (**E**) Quantification of the center crossing times. (**F**) Illustration of the forced swimming test. (**G**) Decreased immobility time of forced swimming test in HMX groups. *n* = 10 per group. All data are presented as mean ± SEM. * *p* < 0.05, ** *p* < 0.01, *** *p* < 0.001, ns, no significant difference.

An open field test was conducted to assess locomotor activity and anxiety-like behaviors in mice exposed to HMX. As shown in Figure 1A,B, the total distance traveled was significantly increased in the medium-dose (100 mg/kg, *p* < 0.01) and high-dose (250 mg/kg, *p* < 0.01) HMX groups compared to the control group, indicating enhanced spontaneous locomotor activity. Furthermore, HMX-treated mice exhibited a dose-dependent increase in central area exploration, as evidenced by significantly increased central distance traveled (medium-dose: *p* < 0.05, high-dose: *p* < 0.01) and percentage of central distance traveled (high-dose: *p* < 0.001) (Figure 1C,D). Moreover, HMX groups showed more central entries compared to the control group (medium-dose: *p* < 0.05, high-dose: *p* < 0.01) (Figure 1E). No significant differences were observed in the low-dose (40 mg/kg) group for any of these parameters.

The forced swimming test was performed to assess despair-like behavior. As shown in Figure 1F,G, the immobility time was significantly reduced in the HMX group compared to the control group (medium-dose: *p* < 0.05, high-dose: *p* < 0.01), while no significant changes were observed in the low-dose groups. Collectively, these results demonstrate that subacute HMX exposure induces hyperactivity and reduces anxiety-like and despair-like behaviors in mice in a dose-dependent manner.

### 3.2. HMX Impairs Hippocampus-Dependent Spatial Learning and Memory

**Figure 2:**
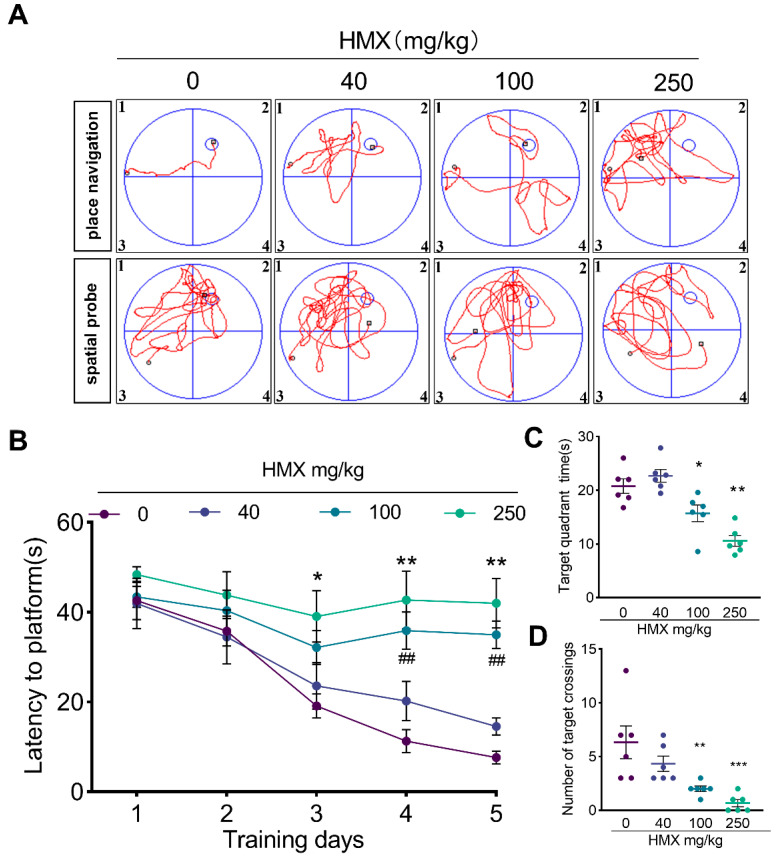
Impaired learning and memory capacity in HM-treated mice. (**A**) Representative traces of mice during place navigation phase (day 1 to 5) and spatial probe phase (day 6) in Morris water maze, red lines represent the swimming trajectories of animals, and blue lines denote the boundaries of the four quadrants. (**B**) Time of latency to platform throughout the place navigation phase. (**C**) HMX groups showed a significant reduction in time spent in target quadrant during spatial probe phase. (**D**) Decreased number of crossings in target quadrant during spatial probe phase of HMX groups. *n* = 6 in each group. All data are presented as mean ± SEM. * *p* < 0.05, ** *p* < 0.01 and *** *p* < 0.001 (vs. 0 mg/kg), ^##^ *p* < 0.001, (100 mg/kg vs. 0 mg/kg), one-way ANOVA.

To investigate the effects of HMX exposure on cognitive function, we performed the Morris water maze test. During the 5-day place navigation phase, all groups showed a progressive decrease in platform latency as training progressed, indicating that mice were able to learn the location of the platform (Figure 2A,B). However, HMX-exposed mice exhibited significantly longer escape latencies compared to control mice. Specifically, the high-dose group showed significantly longer escape latencies on days 3, 4, and 5 (all *p* < 0.05), while the medium-dose group showed significant differences on days 4 and 5 (both *p* < 0.01).

During the spatial probe phase, spatial memory was assessed using a probe trial with the platform removed. As shown in Figure 2C,D, HMX-treated mice spent significantly less time in the target quadrant (medium-dose: *p* < 0.05, high-dose: *p* < 0.01) and made a significant reduction in number of platform crossings (medium-dose: *p* < 0.01, high-dose: *p* < 0.001) compared to control mice. These results indicate that subacute HMX exposure impairs hippocampus-dependent spatial learning and memory in mice.

### 3.3. HMX Induces Hippocampal Demyelination Without Overt Neuronal Necrosis

**Figure 3:**
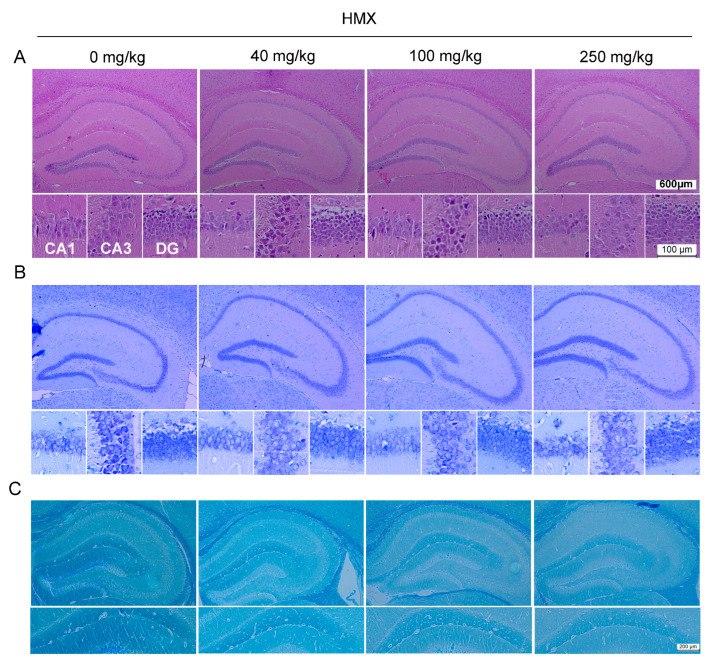
Typical pathological examinations of hippocampus in mice. (**A**) HE. (**B**) Nissl staining in hippocampus, scalebar = 200 μm (up) or 100 μm (down). (**C**) LFB staining. Scalebar = 200 μm.

The abnormal behavioral results indicated that HMX exposure reduced anxiety-like and despair-like behaviors, and impaired memory capacity. Given the critical role of the hippocampus in spatial memory and contextual emotional regulation, we focused our histopathological examination on the hippocampus. Surprisingly, no obvious neuronal degeneration, necrosis, or loss was observed in any of the HMX-treated groups in HE or Nissl staining (Figure 3A,B). LFB staining was then used to assess myelin sheath morphology. As shown in Figure 3C, that LFB-staining intensity was reduced in the medium- and high-dose HMX groups, compared to the control group, indicating a decrease in myelin content. These results demonstrate that subacute HMX exposure induces demyelination in mice without overt neuronal necrosis in the hippocampus.

### 3.4. HMX Reduces the Immunofluorescence Intensity of Myelin Structural Proteins in CA1, CA3 and DG Hippocampal Subregions

**Figure 4:**
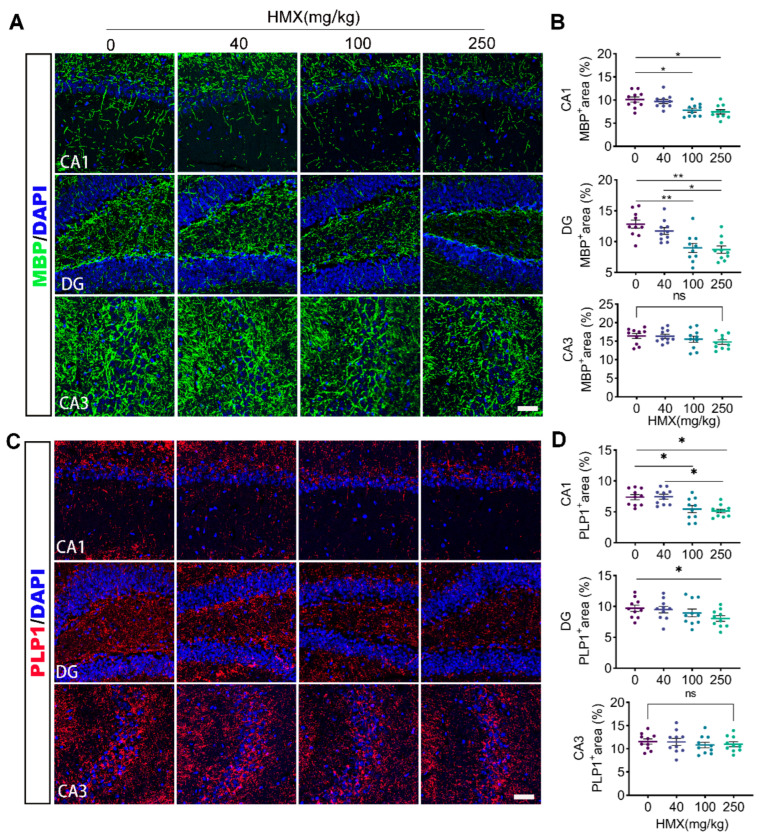
HMX induced decreased levels of MBP and PLP1 in mice. (**A**) Confocal immunofluorescent analysis of MBP in hippocampus. (**B**,**D**) Quantification of the MBP- or PLP1-positive area (percentage of ROI area occupied by specific immunofluorescence signal above threshold) in hippocampal subregions. (**C**) Representative images of immunofluorescence staining for PLP1 in the hippocampus. Scalebar = 25 μm. All data are presented as mean ± SEM. * *p* < 0.05, ** *p* < 0.01, ns, no significant difference, one-way ANOVA.

The structural integrity of myelin is primarily dependent on the normal expression of myelin-related proteins. To further confirm the demyelination observed by LFB staining in the hippocampus, we performed immunofluorescence staining for two major myelin structural proteins: myelin basic protein (MBP) and proteolipid protein (PLP1). MBP, a core structural protein of myelin, helps maintain myelin sheath stability and integrity, which serves as a reliable indicator of myelin damage [17]. PLP1 is critical for myelin formation and maintenance [18]. As shown in Figure 4A,B, MBP immunofluorescence intensity was significantly reduced in the CA1 and dentate gyrus (DG) subregions of the hippocampus in the medium- and high-dose HMX groups compared to the control group (*p* < 0.05). However, no significant change in MBP immunofluorescence intensity was observed in the CA3 subregion. For PLP1, we observed a clear downregulation across CA1 and DG (Figure 4C,D). The high-dose group showed significant PLP1 reduction in CA1 and DG (*p* < 0.05), while the medium-dose group showed a significant decrease only in CA1 (*p* < 0.05). Together, these results provide molecular evidence for HMX-induced hippocampal demyelination.

### 3.5. HMX Decreases the Immunofluorescence Intensity of Oligodendrocyte-Associated Proteins in the CA1, CA3 and DG Hippocampal Subregions

Since the formation, maintenance, and repair of myelin sheaths depend on oligodendrocytes, we further examined the 2′,3′-cyclic nucleotide 3′-phosphodiesterase (CNPase) and oligodendrocyte transcription factor 2 (OLIG2) to clarify the mechanism underlying HMX-induced demyelination. As a specific biomarker of oligodendrocytes, CNPase participates in myelin synthesis and stabilization, and its expression directly reflects the functional status of oligodendrocytes and myelin integrity [19]. Moreover, OLIG2 acts as a pivotal transcription factor during oligodendrocyte development and maturation [20].

**Figure 5:**
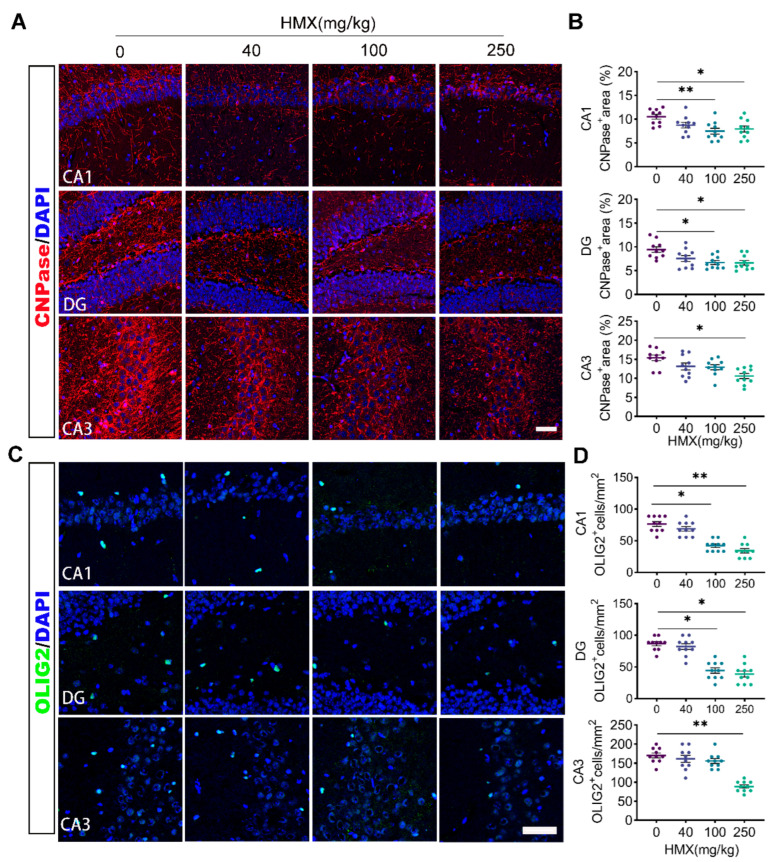
HMX induced downregulation of OLIG2 and CNPase in mice. (**A**) Immunofluorescent analysis for CNPase in hippocampus. (**B**) Quantification of the CNPase positive area in (**A**). (**C**) Representative images of immunofluorescence staining for oligodendrocyte progenitor cell marker OLIG2 in the hippocampus. (**D**) Quantification of OLIG2 in (**C**). Scalebar = 25 μm. All data are presented as mean ± SEM. * *p* < 0.05, ** *p* < 0.01, one-way ANOVA.

Immunofluorescence staining revealed that CNPase immunofluorescence intensity was significantly decreased in CA1 (medium-dose: *p* < 0.01; high-dose: *p* < 0.05), DG (medium-dose: *p* < 0.05; high-dose: *p* < 0.05), and CA3 (high-dose: *p* < 0.05) of the HMX groups compared to the control group (Figure 5A,B). Similarly, OLIG2 immunofluorescence intensity was downregulated in CA1 (medium-dose: *p* < 0.05; high-dose: *p* < 0.01), DG (medium-dose: *p* < 0.05; high-dose: *p* < 0.05), and CA3 (high-dose: *p* < 0.01), while no significant change was found in the low-dose group. The corresponding separate-channel images for DAPI and OLIG2 are included as Supplementary Figure S1. These results suggest that HMX inhibits oligodendrocyte maturation by downregulating OLIG2 expression.

### 3.6. Proteomic Analysis Reveals Global Molecular Changes in the Hippocampus Following HMX Exposure

**Figure 6:**
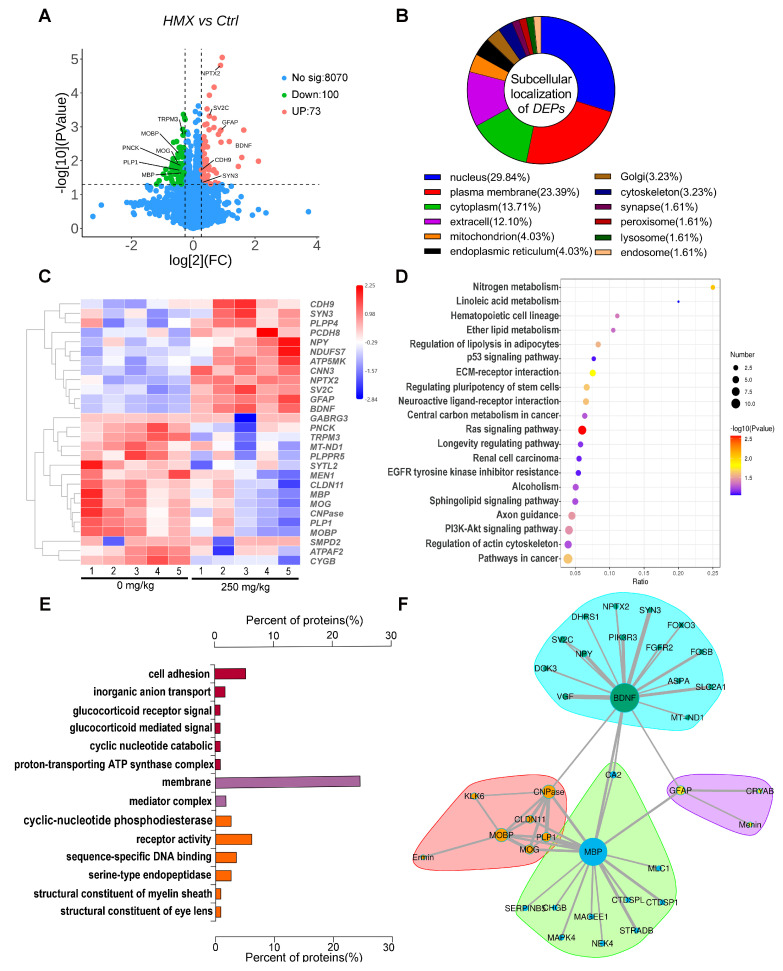
Label–free proteomic analysis of hippocampus in control and HMX–treated mice. (**A**) Volcano plot of the 173 differentially expressed proteins (DEPs) in hippocampus in control and HMX mice. (**B**) Subcellular localization of the DEPs. (**C**) Heat map of the DEPs. (**D**) KEGG pathway enrichment analyses of DEPs in hippocampus. (**E**) GO pathway enrichment analyses were performed for upregulated and downregulated DEPs in hippocampus, lines between protein nodes indicate evidence-based protein-protein interactions, with line thickness reflecting the combined interaction confidence score. (**F**) PPI (protein–protein interaction analysis) analysis of these DEPs was performed. *n* = 5 per group.

To further comprehensively understand the molecular mechanism underlying HMX-induced neurotoxicity, we performed label-free quantitative proteomic analysis on hippocampal tissues from control and high-dose HMX mice. A total of 8243 proteins were identified, of which 173 were differentially expressed (100 upregulated and 73 downregulated) according to the criteria of fold change > 1.5 and *p* < 0.05 (Figure 6A). Subcellular localization analysis showed that the DEPs were predominantly localized in the nucleus (29.84%) and plasma membrane (23.39%), with smaller fractions in the cytoplasm (13.71%), extracellular space (12.10%), mitochondria (4.03%), and synapse (1.61%) (Figure 6B). Notably, numerous nervous system-related proteins were among the DEPs, including myelin-associated proteins (MBP, PLP1, CNPase, MOBP, MOG), and neuronal proteins involved in synaptic function (BDNF, SYN3, SV2C) (Figure 6C). These findings are consistent with our immunohistochemical results and further confirm the effects of HMX on myelination. EGG pathway analysis showed that the DEPs were significantly enriched in neuroactive ligand–receptor interaction, axon guidance, PI3K-Akt signaling, Ras signaling, and nitrogen metabolism pathways (Figure 6D). GO functional enrichment analysis revealed that the DEPs were primarily enriched in biological processes related to nervous system development, synaptic transmission, myelin sheath organization, and neuroactive ligand–receptor interaction (Figure 6E). Next, protein–protein interaction (PPI) network analysis indicated that the core DEPs formed interconnected clusters associated with myelin and neuronal function. Importantly, almost all myelin-related proteins identified in this study, including MBP, PLP1, CNPase, MOBP, and MOG, were significantly downregulated, providing strong proteomic evidence for HMX-induced demyelination, while SV2C and BDNF were involved in regulating synaptic transmission and neuronal survival (Figure 6F).

## 4. Discussion

Energetic materials are indispensable for modern industrial and military applications, but occupational exposure poses significant health risks. Among nitramine explosives, RDX and CL-20 have well-characterized neurotoxic profiles, primarily manifesting as acute seizures and cognitive deficits. However, the mechanisms underlying HMX-induced central nervous system (CNS) damage remain poorly defined. In this study, we provide the first comprehensive evidence that subacute HMX exposure induces neurobehavioral deficits and hippocampal demyelination in mice. Our findings reveal a novel mechanism of HMX neurotoxicity that is distinct from other nitramine explosives and have important implications for occupational health protection of HMX.

A striking finding of our study is that HMX exposure reduced anxiety-like and despair-like behaviors in mice, which contrasts with the increased anxiety and depressive-like phenotypes induced by most neurotoxicants. However, similar behavioral changes have been consistently reported in RDX-exposed rodents, which also exhibit hyperactivity and reduced anxiety [21]. This shared behavioral phenotype suggests that nitramine explosives may modulate common neural circuits involved in emotional regulation. The reduced anxiety and despair-like behaviors observed here are unlikely to represent an “antidepressant-like” effect, but rather reflect abnormal emotional processing caused by CNS damage. Indeed, epidemiological studies have shown that HMX-exposed workers exhibit a range of emotional disturbances, including irritability, emotional lability, and apathy. The hippocampus cooperates with the amygdala and prefrontal cortex to modulate contextual emotional processing, and hippocampal demyelination has been shown to disrupt inter-regional neural circuit connectivity and induce abnormal emotional behaviors [22,23]. Future studies using electrophysiological and optogenetic approaches are needed to elucidate how hippocampal demyelination specifically affects emotional processing circuits.

Consistent with previous epidemiological findings, our results demonstrate that HMX exposure impairs hippocampus-dependent spatial learning and memory. The Morris water maze test is a gold standard for assessing hippocampal function, and the observed deficits in both acquisition and retention phases indicate that HMX disrupts multiple stages of memory formation [24]. Importantly, we found that these cognitive deficits occurred in the absence of overt neuronal necrosis, as evidenced by normal H&E and Nissl staining. This is a crucial observation that distinguishes HMX neurotoxicity from many other neurotoxicants, which primarily act by inducing neuronal death. Instead, our results clearly show that HMX targets the myelin sheath, leading to significant hippocampal demyelination. Myelin is essential for rapid saltatory conduction of nerve impulses and for maintaining axonal integrity [25,26,27]. Disruption of myelin integrity impairs neural communication between brain regions, which can lead to cognitive dysfunction even in the absence of neuronal loss [26]. This mechanism may explain the persistent cognitive deficits observed in HMX-exposed workers, as demyelination can lead to long-term neural circuit dysfunction. Oligodendrocytes are the myelinating cells of the CNS, and their development is tightly regulated by the transcription factor OLIG2 [28,29]. OLIG2 is essential for the specification, proliferation, and differentiation of oligodendrocyte progenitor cells (OPCs) into mature myelinating oligodendrocytes [30]. We found that HMX exposure was associated with reduced immunofluorescence intensity of OLIG2 in all hippocampal CA1, CA2 and DG subregions, which was accompanied by decreased immunofluorescence signal for the mature oligodendrocyte marker CNPase and myelin structural proteins MBP and PLP1. This pattern is consistent with, though not definitive proof of, the interpretation that HMX may inhibit OPC differentiation into mature oligodendrocytes, leading to reduced myelin production and subsequent demyelination. This interpretation is supported by previous studies showing that OLIG2 deficiency results in hypomyelination and cognitive deficits in animal models [31,32,33], and by our proteomic data showing convergent downregulation of multiple myelin-related proteins. Interestingly, we note an apparent discrepancy between our immunofluorescence and proteomic findings for OLIG2: immunofluorescence quantification demonstrated a significant reduction in OLIG2-positive cells within the CA1, CA3, and DG hippocampal subregions, whereas bulk proteomic analysis of whole hippocampal homogenates detected no statistically significant change in total OLIG2 protein abundance. This divergence does not represent conflicting results; instead, it stems from inherent differences in sampling scope and analytical modality between the two approaches.

Immunofluorescence allows targeted, single-cell-resolution quantification of OLIG2-expressing cells restricted to the three examined subregions. By contrast, proteomic profiling captures total OLIG2 protein levels across the entire hippocampus, including subregions not assessed in our immunofluorescence assays such as CA2. Localized decreases in OLIG2-positive cell density can therefore be diluted in bulk tissue homogenates, potentially falling below the quantitative dynamic range of mass spectrometry. Accordingly, these two methods offer complementary rather than opposing insights: immunofluorescence directly demonstrates cell- and region-specific phenotypic alterations, while proteomics provides an unbiased survey of global molecular changes across the whole tissue.

Our proteomic analysis provides further supportive evidence for this mechanism and reveals additional molecular pathways involved in HMX neurotoxicity. Consistent with our immunofluorescence results, almost all myelin-related proteins identified in the proteomic analysis were significantly downregulated, providing convergent, unbiased evidence for HMX-induced demyelination. In addition to myelination pathways, we found significant enrichment of DEPs in synaptic transmission and neuroactive ligand–receptor interaction pathways. The downregulation of key synaptic proteins such as BDNF, SYN3, and SV2C suggests that HMX also disrupts synaptic function, which may act synergistically with demyelination to exacerbate neurobehavioral deficits. BDNF is a critical neurotrophic factor that regulates synaptic plasticity, neuronal survival, and oligodendrocyte development [34].

Another interesting finding from our proteomic analysis is the enrichment of DEPs in the nitrogen metabolism pathway. HMX contains four nitro groups, and its metabolism in the body can generate reactive nitrogen species (RNS) and other toxic intermediates. The enrichment of nitrogen metabolism pathway proteins provides direct evidence that HMX crosses the blood–brain barrier and undergoes biotransformation in the brain. The generation of RNS in the brain could lead to oxidative stress and nitrosative damage, which may contribute to oligodendrocyte dysfunction. Oligodendrocytes are particularly vulnerable to oxidative stress due to their high metabolic rate and low antioxidant capacity [35,36]. This vulnerability may explain why HMX preferentially targets oligodendrocytes rather than neurons at the doses tested.

It is important to compare the neurotoxic mechanisms of HMX with those of other nitramine explosives to understand their differential health effects. RDX primarily induces acute epileptic seizures by directly blocking GABA_a_ receptors, with relatively little evidence of chronic demyelinating damage [37,38]. In contrast, our results show that HMX causes more persistent CNS damage by targeting oligodendrocytes and inducing demyelination. This difference may be due to their distinct chemical structures and metabolic profiles. Additionally, HMX may have a higher affinity for molecular targets involved in oligodendrocyte development, such as OLIG2 or its upstream regulators.

While this study provides novel insights into HMX neurotoxicity, several limitations should be acknowledged. First, we used a 28-day subacute exposure model with relatively high doses of HMX, which may not fully replicate the long-term low-dose exposure characteristics of occupational populations. Future studies using chronic low-dose exposure models are needed to evaluate the long-term neurological effects of HMX. Mechanistically, our data suggest that HMX exposure is associated with downregulation of OLIG2 expression in the hippocampus, accompanied by decreased expression of the mature oligodendrocyte marker CNPase and myelin structural proteins MBP and PLP1. This pattern is consistent with the interpretation that HMX may inhibit oligodendrocyte maturation, leading to reduced myelin production and subsequent demyelination. However, we acknowledge that additional studies (such as in vitro OPC differentiation assays or OLIG2 overexpression experiments) would be necessary to definitively establish a causal relationship. Third, we focused exclusively on the hippocampus in this study, as this region is critically involved in the behavioral phenotypes we observed (spatial learning and memory, and emotional regulation). However, HMX may also affect other brain regions such as the corpus callosum, cortex, and cerebellum. Future studies should investigate the regional specificity of HMX-induced demyelination to provide a more comprehensive understanding of its neurotoxic profile. Finally, our study was conducted in male mice only, and it is important to determine whether female mice exhibit similar or different susceptibility to HMX neurotoxicity.

In conclusion, our study demonstrates that subacute HMX exposure induces hippocampal demyelination and neurobehavioral deficits in mice, accompanied by decreased expression of the mature oligodendrocyte marker CNPase and myelin structural proteins MBP and PLP1. These findings provide a novel mechanistic insight into HMX neurotoxicity and highlight the importance of considering demyelination as a key endpoint in the risk assessment of nitramine explosives. From a public health perspective, our results suggest that occupational HMX exposure may increase the risk of demyelinating disorders and cognitive impairment, underscoring the need for improved protective measures for workers exposed to HMX. Future studies should focus on developing biomarkers of HMX-induced demyelination and identifying potential therapeutic targets for the prevention and treatment of HMX-induced neurological damage.

## 5. Conclusions

Subacute exposure to HMX induces dose-dependent neurobehavioral abnormalities in mice, including hyperactivity, reduced anxiety-like and despair-like behaviors, and impaired hippocampus-dependent spatial learning and memory. These deficits are associated with significant hippocampal demyelination, which occurs in the absence of overt neuronal necrosis. Mechanistically, HMX exposure is associated with downregulation of OLIG2 expression in the hippocampus, accompanied by decreased expression of the mature oligodendrocyte marker CNPase and myelin structural proteins MBP and PLP1. Proteomic analysis further reveals that HMX disrupts multiple biological processes in the hippocampus, including myelination, synaptic transmission, and neuroactive ligand–receptor interaction. These findings provide a novel mechanistic understanding of HMX neurotoxicity and have important implications for the development of occupational health protection strategies against HMX exposure.

## Data Availability

The original contributions presented in this study are included in the article/Supplementary Material. Further inquiries can be directed to the corresponding authors (Zhiyong Liu, lyz30302@126.com).

## Supplementary Materials

The following supporting information can be downloaded at: https://www.mdpi.com/article/10.3390/toxics14070605/s1, Figure S1: Representative images of immunofluorescence staining for oligodendrocyte progenitor cell marker OLIG2 in the hippocampus. Scalebar = 25 μm. Green = OLIG2, blue = DAPI. CA1 (Cornu Ammonis 1), CA3 (Cornu Ammonis 3), DG (Dentate Gyrus).
