# Supplementary material for: Subacute Octahydro-1,3,5,7-tetranitro-1,3,5,7-tetrazocine Exposure Induces Neurobehavioral Deficits and Hippocampal Demyelination in Mice
Source: Toxics. 2026 Jul 11;14(7):605. doi: 10.3390/toxics14070605 (PMC13431414; doi:10.3390/toxics14070605)
Supplement: Supplementary file 1 [file toxics-14-00605-s001.zip › toxics-4406074-supplementary.pdf]

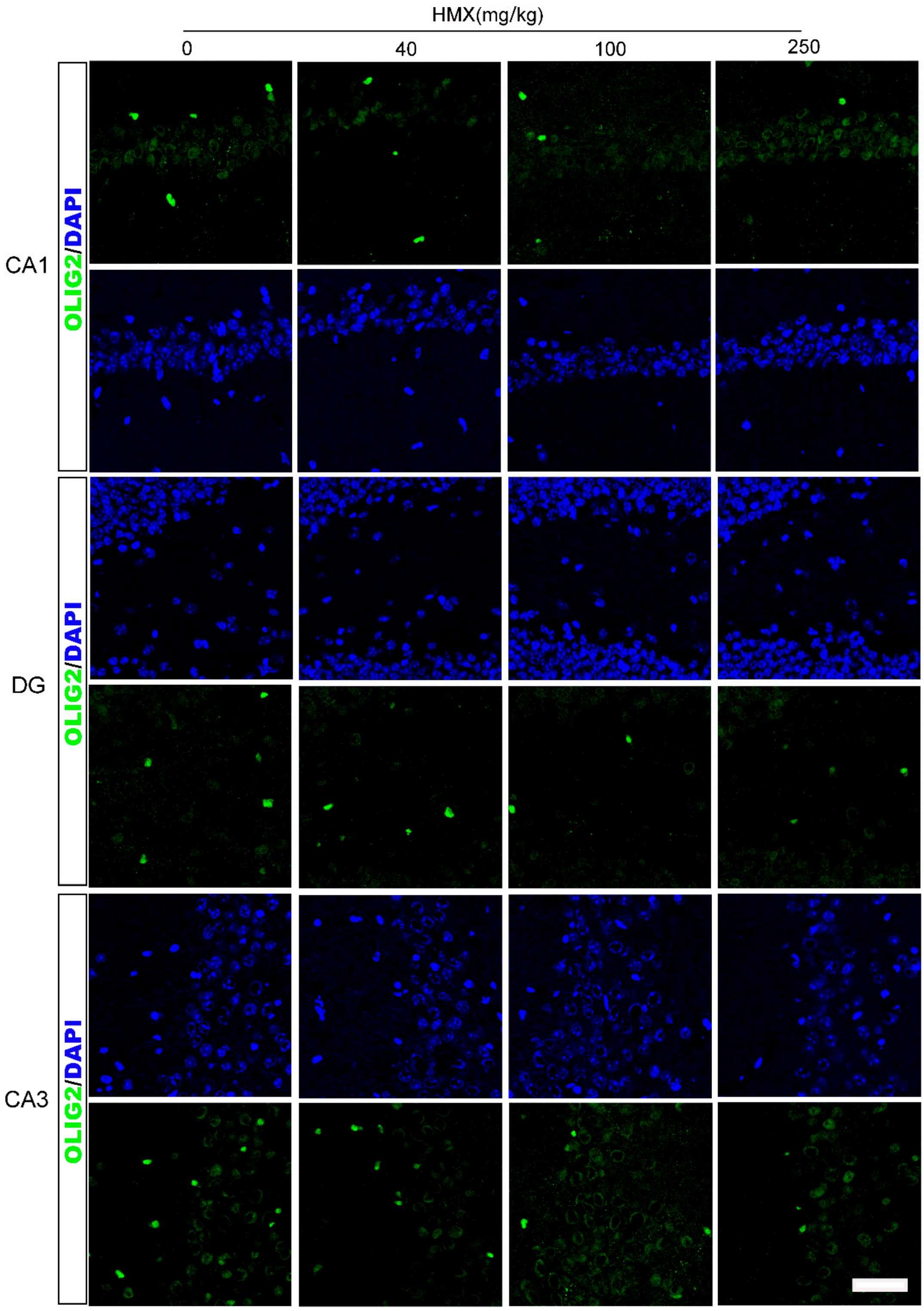

Supplementary Figure S1： Representative images of immunofluorescence staining for oligodendrocyte progenitor cell marker OLIG2 in the hippocampus. Scalebar = 25 μm. Green = OLIG2, blue = DAPI. CA1(Cornu Ammonis 1), CA3(Cornu Ammonis 3), DG (Dentate Gyrus).
